# Precise Electrode Co‐Alignment in Deep Brain Stimulation Fusing Neuroimaging and Electrophysiology

**DOI:** 10.1111/ejn.70309

**Published:** 2025-11-19

**Authors:** Igor Varga, Daniel Novak, Dusan Urgosik, Jan Kybic, Filip Ruzicka, Pavel Filip, Robert Jech, Andreas Horn, Eduard Bakstein

**Affiliations:** ^1^ Department of Cybernetics Faculty of Electrical Engineering, Czech Technical University Prague 2 Czech Republic; ^2^ Czech Centre for Phenogenomics BIOCEV—Institute of Molecular Genetics Prague Czech Republic; ^3^ Department of Neurology, First Faculty of Medicine Charles University and General University Hospital in Prague Prague Czech Republic; ^4^ Center for Magnetic Resonance Research (CMRR) University of Minnesota Minneapolis Minnesota USA; ^5^ Institute for Network Stimulation, Clinic for Stereotaxy and Functional Neurosurgery University Hospital Cologne Cologne Germany; ^6^ Center for Brain Circuit Therapeutics Department of Neurology Brigham & Women's Hospital, Harvard Medical School Boston Massachusetts USA; ^7^ MGH Neurosurgery & Center for Neurotechnology and Neurorecovery (CNTR) at MGH Neurology Massachusetts General Hospital, Harvard Medical School Boston Massachusetts USA; ^8^ National Institute of Mental Health Klecany Czech Republic

**Keywords:** deep brain stimulation, electrophysiology, neuroimaging, optimisation framework, precise electrode localisation, subthalamic nucleus

## Abstract

We present a multimodal framework to improve the precision of electrode placement in deep brain stimulation (DBS) by fusing preoperative neuroimaging with intraoperative electrophysiology for accurate electrode co‐alignment. The workflow integrates automated subthalamic nucleus (STN) segmentation from preoperative MRI using a two‐step convolutional neural network (CNN), classification of microelectrode recordings (MER) with a transformer encoder and spatial co‐alignment via a discrete optimisation procedure. Implemented as a 3D Slicer plugin, the pipeline enables real‐time visualisation and interactive use during surgery. In validation on retrospective data of 17 trajectories from 12 Parkinson's disease patients, co‐alignment reduced the mean lateral localisation error by 0.3 mm relative to an intraoperative reference, indicating improved agreement between electrophysiological and anatomical targets. Automated STN segmentation achieved a Dice similarity of 0.62 ± 0.10, providing a robust starting point for manual refinement. This approach improves the understanding of electrode position within STN during surgery, incorporating preoperative and intraoperative data, offers clinicians a practical, real‐time tool to enhance targeting accuracy. By directly integrating imaging and MER evidence, the framework addresses persistent challenges in DBS and represents a step toward more personalised and precise neurosurgical interventions.

**Abbreviations**
CNN: convolutional neural network
CT: computed tomography
DBS: deep brain stimulation
MER: microelectrode recordings
MNI: Montreal Neurological Institute
MRI: magnetic resonance imaging
NRMS: normalised root mean square
OARM: intraoperative O‐arm imaging
PCA: principal component analysis
PD: Parkinson's disease
ROI: region of interest
STN: subthalamic nucleus

## 1 Introduction

Integrating information obtained from neuroimaging data with electrophysiological recordings is a common approach for electrode placement in deep brain stimulation (DBS), particularly for the treatment of neurological disorders such as Parkinson's disease (PD) (Guo et al. 2007). Accurate localisation of the subthalamic nucleus (STN), a primary target of PD DBS, during surgery is important to maximise clinical outcomes. However, its small size, deep location and individual anatomical variability pose significant challenges for precise identification and segmentation (Kocabicak et al. 2012; Keuken et al. 2014).

Typically, the STN is first localised using preoperative magnetic resonance imaging (MRI). The imaging is then used for surgical planning, which determines the intended trajectory of the DBS stimulation electrode. While indispensable, neuroimaging techniques have limitations in spatial resolution and may introduce distortions, particularly in deep brain structures such as the STN (Dormont et al. 2004). Accurate localisation of the STN is further complicated by its low contrast from substantia nigra, even if specialised sequences such as T2 or T2* are used (Schafer et al. 2012).

The actual position of the STN during subsequent DBS implantation may deviate from the surgical plan due to several factors, namely stereotactic and co‐registration inaccuracies, air entering the skull due to cerebrospinal fluid leakage (pneumocephalus) or postural changes. These aspects may lead to brain shift or non‐linear brain deformations, complicating the accurate implantation (Zrinzo 2019; Park et al. 2018).

For these reasons, many centres use intraoperative invasive microelectrode recordings (MER), which can provide corrective information for deviations from the imaging‐based surgical plan and provide a more accurate STN delineation before determining the final lead location (Montgomery 2012).

MER has long been instrumental in confirming or refining the optimal target site for electrodes that are entered during surgery. Typically, multiple MER electrodes are entered to probe a larger field of anatomical tissue, and this approach improves motor outcomes in Parkinson's disease, especially for symptoms such as tremor and rigidity (Temel et al. 2007). However, the success of MER in clinical practice depends on signal quality, and requires manual inspection and integration by experts with extensive training in the operating room (Gross et al. 2006).

Together, these challenges, issues in imaging accuracy when used alone and the complicated nature of understanding MER signals, highlight the need for advanced multimodal approaches that integrate electrophysiological information obtained in the operating room and neuroimaging data collected preoperatively. Discrepancies observed during the merging of the two sources of information can provide clues about the brain change and valuable insights when determining the final implantation position of the permanent lead. Indeed, such discrepancies do occur: studies report that MRI can miss vital anatomical details of the STN in up to 20% of cases (Lozano et al. 2018), and different field strengths can shift the apparent boundaries of the STN (Verhagen et al. 2016), notably leaving the dorsal margin inconsistently defined. Although high‐field magnetic resonance imaging (7T) can reduce lateral border ambiguities, the dorsal boundary remains challenging to pinpoint precisely. In this context, MER still offers critical supporting information, and optimising its integration with imaging is an active area of research aiming to minimise brain shift effects and other surgical uncertainties.

A recent key advancement in this direction is the Lead‐OR platform (Oxenford et al. 2022), which improves DBS targeting via real‐time spatial display of MER signals within the MRI‐defined stereotactic space (and segmented nuclei). Here, we build upon and significantly extend this platform by proposing an end‐to‐end pipeline capable of automatically enhancing electrode localisation by integrating deep learning‐based STN segmentations with MER co‐alignments. Convolutional neural networks (CNN) have shown strong performance in segmenting subcortical structures, including STN (Manjón et al. 2020; Baniasadi et al. 2022). Our approach employs a two‐step CNN model for precise anatomical segmentation, followed by an optimisation framework that aligns MER signals within the segmented STN. Implemented as a 3D Slicer (Fedorov et al. 2012) plugin and integrated into Lead‐OR, this pipeline provides a robust framework for clinicians and researchers to simultaneously visualise and analyse neuroimaging and electrophysiological data. By enhancing the understanding of electrode positioning in real‐time, our method aims to support more accurate targeting during surgery and ultimately improve patient outcomes in DBS.

## 2 Methods

**FIGURE 1:**
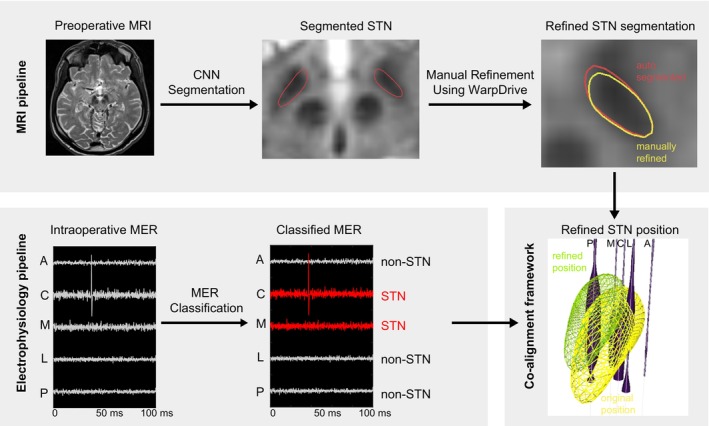
Overview of the DBS electrode co‐alignment framework. This framework combines preoperative MRI with intraoperative MER data, providing enhanced realtime information about electrode positions at any moment during surgery. Top row: The subthalamic nucleus (STN) is segmented automatically (red) from pre‐operative MRI data, with optional manual refinements applied to enhance anatomical accuracy (yellow). Bottom row: Intraoperative MER signals from parallel electrodes (AAnterior, C‐Central, M‐Medial, L‐Lateral, P‐Posterior) are automatically classified into inside/outside STN groups. The co‐alignment framework then aligns the individual STN contour (yellow) with the MER classifications and finds the likely shift leading to a refined STN position (green), w.r.t. the MER electrodes (dark blue, cylinder width represents the NRMS value at each recording position).

Our aim is to provide surgeons with real‐time information on the position of the electrode relative to surrounding anatomical structures during surgery. Ideally, this process should achieve maximum precision and operate in real time. For the purpose of this article, we refer to this process as ‘electrode location’, which should not be confused with similar terms used postoperatively, i.e., in the process of localising electrodes already implanted based on postoperative imaging. In summary, to accurately localise electrodes in real time during DBS surgery, we implemented a shift estimation method that combines deep learning with classical optimisation techniques. This method integrates neuroimaging and electrophysiological data within a structured pipeline, implemented as a 3D Slicer extension. The workflow, outlined in Figure 1, can be broken down into the following components:

1. STN Segmentation from preoperative MRI: We developed a two‐step CNN model capable of automatically localising and segmenting the STN from preoperative MRI scans. This approach generates a 3D surface mesh representation of the STN in stereotactic space. This automated segmentation can be manually refined using published tools available in the Lead‐DBS framework (Oxenford et al. 2024).
2. Classification of MER signals: To address the second requirement, we developed a transformer‐based encoder model that processes microelectrode recording (MER) signals, extracting electrophysiological features essential for STN localisation and classifying recording sites as either within or outside the STN.
3. Co‐alignment through discrete optimisation: To integrate these two sources of information, the segmented STN shapes and classified MER data were combined within an optimisation framework designed to refine electrode placement, thereby compensating for potential brain displacement such as intraoperative brain shift.

### 2.1 Data and Labelling

Five data subsets from three data sources were used to train and validate the different steps of the method, summarised in Table 1.

**TABLE 1:** Datasets used for training and testing of segmentation, microelectrode signal classification and co‐alignment.

Usage	Data source	Size and composition	Modalities	Notes
Segmentation train	OASIS‐3	1366 selected subjects	T1, T2, pBrain automatic labels	OASIS‐3 contains various conditions including neurodegenerative
Segmentation test	OASIS‐3	10 subjects manually labelled	T1, T2, Manual labels	Manual labels by a trained expert
	DISTAL (IXI sub‐set)	22 subjects manually labelled	T1, T2, Manual labels	Manual labels by Ewert (Ewert et al. 2018)
MER train	PRAGUE‐MER	112 PD patients, 935 microelectrodes	MER, MER labels	MER signals labelled intraoperatively by a neurologist
MER test	PRAGUE‐mutlimodal	30 PD patients	T1, T2, MER	Subjects without STN recordings excluded; total 18 subjects, 28 sides
Co‐alignment test	PRAGUE‐multimodal	12 PD patients	T1, T2, MER, OARM	1 subject excluded; 4 missing unilateral MER/MRI; 17 STNs in total

#### 2.1.1 OASIS‐3 MRI Dataset

We used the publicly available OASIS‐3 dataset (LaMontagne et al. 2019) to train and evaluate our segmentation model. OASIS‐3 includes high‐resolution structural magnetic resonance imaging, along with cognitive and clinical evaluations from 1378 participants, which include individuals who are cognitively healthy, have mild cognitive impairment (MCI) or are diagnosed with Alzheimer's disease (AD).

For this study, we focussed on T1 and T2‐weighted MRI scans acquired using 1.5T and 3T MRI scanners. The dataset includes individuals aged 42–95 years. We trained our CNN model using segmentation labels generated by pBrain (Manjón et al. 2020). For validation, the STN in the test set was manually segmented using ITK‐SNAP (Yushkevich et al. 2006).

#### 2.1.2 External Validation: IXI Dataset

For external validation of our MRI segmentation model, we used a manually labelled subset of the IXI dataset, which was previously used to develop the DISTAL atlas (Ewert et al. 2018). This dataset includes T1‐ and T2‐weighted MRI scans from individuals without known health conditions, acquired using 1.5T and 3T MRI scanners.

A cohort of 22 individuals (14 women, 8 men) aged 55–70 years (mean 63.8 ± 4.3 years) was selected to align with clinical populations seen in DBS. The STNs were manually segmented, primarily using T2‐weighted images for better accuracy. These manual labels, including those for adjacent structures, were supplied by the original authors. A detailed description of the labelling procedure is available in Ewert et al. (2018).

#### 2.1.3 PRAGUE‐MER Dataset

The PRAGUE‐MER dataset was used to train and evaluate the MER classification model. It contains MER recordings collected from 112 patients with PD who underwent DBS surgery.

MER recordings were performed using one to five tungsten microelectrodes in a cross configuration, with a 2 mm spacing between the electrodes. The electrodes advanced by 0.5 mm between the recording positions. Ten‐second recordings were collected at each position using the Leadpoint (Medtronic, MN) system, sampled at 24 kHz and bandpass filtered between 500 and 5000 Hz.

Each recording position was manually labelled by the surgical team as STN or non‐STN, forming the basis for classification.

#### 2.1.4 Prague Multimodal Dataset (PD Patients Undergoing DBS)

The Prague Multimodal Dataset consists of 18 participants with PD who underwent subthalamic nucleus deep brain stimulation (STN‐DBS). This group included 8 women and 10 men, aged 47–70 years (mean 58.8 ± 7.6 years).

Among these participants, 12 had intraoperative O‐arm CT (OARM) scans, but only 11 were used for co‐alignment validation due to imaging problems. The dataset includes 3T T2‐weighted MRI, 1.5T T1‐weighted MRI, O‐arm CT (OARM) and MER.

MER data were recorded and labelled consistently with the PRAGUE‐MER dataset. Unlike PRAGUE‐MER, this dataset includes both electrophysiological and imaging data, allowing for co‐alignment validation.

### 2.2 Model for Subcortical Segmentation

We used a cascaded neural network architecture for segmentation, dividing the process into two stages. The first stage involves predicting the centre‐of‐mass coordinates of the STN, while the second stage focusses on delineating its boundaries to generate a 3D surface mesh.

#### 2.2.1 MRI Preprocessing

The preprocessing pipeline we employed follows the approach outlined in our previous work (Varga et al. 2024).

To standardise intensity values, we applied fuzzy c‐means clustering combined with white matter intensity normalisation (Reinacher et al. 2019) to ensure intensity consistency between participants. White matter masks were generated using the deep Atropos algorithm (Avants et al. 2011).

To perform segmentation, all brain images were linearly co‐registered to the Montreal Neurological Institute (MNI) space (Grabner et al. 2006). This standardisation was used exclusively for the segmentation model, which requires subjects to be aligned to a common anatomical space. The resulting individual segmentations are then inverse‐transformed to the patient's native space.

#### 2.2.2 STN Centre Detection

The centre detection employs a CNN‐based brain extraction method from ANTs (Isensee et al. 2021), improving segmentation accuracy and processing efficiency. Detailed descriptions of the general preprocessing pipeline can be found in our previous publication (Varga et al. 2024).

A region of interest (ROI) for the STN was defined in the MNI space, extending 3 mm along each axis to account for inter‐subject variability. Symmetry‐based processing mirrored one hemisphere onto the other, allowing both STNs to be processed with a single model.

Within this ROI, a CNN‐based model predicts the centre coordinates of the STN (*x*, *y*, *z*), scaled to a range [0, 1]. The network consists of convolutional blocks with ReLU activation and max‐pooling layers, while a Sigmoid activation in the final layer constrains the output within the normalised range (network scheme is in  Supporting Information S1: Figure S1).

#### 2.2.3 STN Shape Segmentation

The next stage involved the segmentation of the STN. Following the initial estimate of the STN centre, we define a refined ROI around this estimated position. For the segmentation, we then employ a CNN model with architecture similar to that used in centre detection, consisting of convolutional blocks followed by max‐pooling layers.

The network was trained to predict the weights of the principal component analysis (PCA) representing the shape of the STN as standard 3D mesh shape primitives (Varga et al. 2024). This PCA‐based approach provides a compact representation of shape variability around the STN centre, allowing efficient learning and accurate predictions.

To improve segmentation accuracy, we incorporate the Warp Drive toolbox (Oxenford et al. 2024), which allows manual refinement of the STN shape. This allows clinicians to refine automated segmentation, ensuring a more accurate representation of the STN. The adjusted STN shape is then used in the co‐alignment pipeline to improve alignment with recorded electrode positions, reducing errors in shift estimation.

### 2.3 Co‐Alignment of MER Within MRI‐Based STN

In this step, the segmented 3D contour of the STN in patient native space—optionally refined manually and derived from preoperative MRI—is automatically aligned with intraoperative MER data to obtain a more precise estimate of electrode positioning within and around the STN. This alignment process involves two key components: MER signal classification and spatial optimisation of electrode positions.

#### 2.3.1 MER Signal Classification

First, we developed a classification model to identify electrode positions within the STN, based on normalised root mean square (NRMS) values extracted at each recording step from the MER (Moran et al. 2006). NRMS values, adapted from methods used in the Lead‐OR platform (Oxenford et al. 2022), provide a quantitative measure of neuronal activity and are highly effective in characterising STN signals (Thompson et al. 2018).

We implemented a transformer encoder model (Vaswani et al. 2023) to classify microelectrode recordings (MERs) as originating from inside or outside the STN.

The transformer architecture is particularly well suited to modelling sequential dependencies, making it ideal for handling structured MER sequences acquired along each microelectrode trajectory. In contrast to methods that classify each depth independently, the transformer encoder integrates contextual information across multiple recording depths to improve classification robustness.

Our formulation assumes that classification at a given depth *x*_*i*_ may depend on signals from adjacent depths {*x*_*i* − *k*_,…,*x*_*i*_,…,*x*_*i* + *k*_}, without requiring causality constraints (i.e., both past and future inputs are available during classification). The model was trained using cross‐entropy loss on expert‐labelled MER segments.

#### 2.3.2 Electrode‐STN Co‐Alignment

To improve the spatial consistency between intraoperative electrophysiological signals and preoperative imaging, we implemented a co‐alignment framework based on spatial optimisation. The aim is to minimise discrepancies between the MRI‐derived STN mesh and the classification of MER sites as within or outside the STN.

The optimisation problem is formalised using a cost function *C*(*x*,*s*,*α*) that evaluates the quality of a given spatial transformation. Specifically, we estimate a shift vector *s* ∈ *R* ^3^ and an STN scaling factor *α* ∈ *R*+ that improves the alignment between the MER points and the anatomical STN label. Optimisation penalises inconsistencies between MER classification (STN vs. non‐STN) and their spatial inclusion within the transformed STN mesh.

The optimal transformation parameters are obtained by solving:

$$\begin{array}{c} (\hat{s},\hat{\alpha })=\text{argmin}C\left(x,s,\alpha \right)\\ s,\alpha \end{array}$$

Let *x*_*i*_ denote the original coordinates of the *i*‐th MER recording, and let *y*_*i*_ ∈ {0, 1} be its classification label. Let *y*_i_* indicate whether the transformed recording position *x*_*i*_ − *s* is within the scaled mesh *α*M. Then the objective function is defined as:

$$C\left(x,s,\alpha \right)=\frac{1}{N}\sum_{i=1}^{N}\delta \left(y_{i}\neq y_{i}^{*}\right)\cdot d\left(x_{i}-s,\mathrm{\alpha M}\right)$$

where:

- *δ* (*y*_*i*_ ≠ *y*_i_*) is an indicator function that equals 1 when the classification and mesh inclusion disagree (i.e., a misalignment), and 0 otherwise.
- *d*(·*,α*M) denotes the shortest Euclidean distance from the point to the surface of the scaled STN mesh.

Minimising this cost enforces anatomical and electrophysiological overlap. Detailed step‐by‐step explanation of the criterion calculation is provided in Supplementary Information S1: Section S1.3. Powell's optimisation method (Powell 1964) was chosen for its robustness in non‐differentiable optimisation problems, suitable for handling discrete inclusion logic and geometric distance functions. This approach reduces the need for manual MER inspection and interpretation during the DBS procedure and aims to improve anatomical precision, accounting for individual anatomical variability.

We estimate a relative transform between the STN identified in the MRI image and the MER trajectories, defined by surgical plan, to maximise consistency with the observed electrophysiology measured by MER, maintaining plausible limits of brain shift. In our implementation, we apply translation to the electrode trajectories (electrodes are shifted in native space to their most likely position) and isotropic scaling to the STN mesh to adapt for disparity between STN hypointensity and electrophysiological STN. We do not rotate either object. This mixed parameterisation reflects the shifts that may be introduced due to stereotactic inaccuracies (i.e., electrode positions deviate from the plan) or brain shift (i.e., actual position of the STN differs from the preoperative MRI) whereas labelling or normalisation errors and differences in MRI hypointensity and electrophysiological representation of the STN may require modest global scaling of the STN (Schlaier et al. 2011).

The boundaries used for the purpose of the evaluation were ∥t∥ ≤ 3 mm and *s* ∈ [0.8, 1.2] and are user‐adjustable in the GUI provided. We emphasise that only the relative pose, showing electrode position with respect to the STN, is consequential for the selection of permanent lead location.

## 3 Results

The primary outcome of our work is a platform that enables automatic processing and real‐time co‐alignment of MRI and MER data, demonstrated in Supporting Information Video Data S2 and distributed as the *DBSCoalignment* extension for 3D Slicer. We developed multiple extension modules within 3D Slicer to assist surgeons with planning and visualisation tasks. While based on the Lead‐OR pipeline (Oxenford et al. 2022), our implementation significantly extends its functionality by introducing a pipeline capable of estimating the optimal transformation for aligning electrodes within the STN. Below, we provide validation results for the individual processing steps.

### 3.1 STN Shape Segmentation Results

In the first step, we generate a patient‐specific STN shape based on imaging data. The effectiveness of our segmentation approach is shown in Table 2, with Dice similarity scores indicating the agreement between automated and manually refined segmentations. Dice scores of 0.589 (±0.112) for the left STN and 0.619 (±0.102) for the right STN on the OASIS test set, and 0.578 (±0.101) for the left STN and 0.609 (±0.095) for the right STN on the IXI dataset. Detailed distribution plots for both datasets are available in Supporting Information S1: Section S2.2. As seen from the results, the segmentation accuracy is comparable to that of the pBrain model (Manjón et al. 2020) when applied to the dataset used. To ensure accurate shape representation, manual refinement using WarpDrive was applied in the next step when needed to correct discrepancies in automated segmentation. Additional examples of automated segmentation are available in Supporting Information S1: Section S2.1.

**TABLE 2:** Comparison of segmentation accuracy (Dice coefficient) for different methods and datasets.

Method	Data	STN left	STN right
pBrains		0.862	0.867
pBrains (OASIS labels)	3T	0.572 (0.105)	0.583 (0.107)
CNN method (OASIS labels)	3T	0.589 (0.112)	0.619 (0.102)
CNN method (Evert IXI labels)	3T	0.578 (0.101)	0.609 (0.095)

### 3.2 MER Signal Classification Results

**FIGURE 2:**
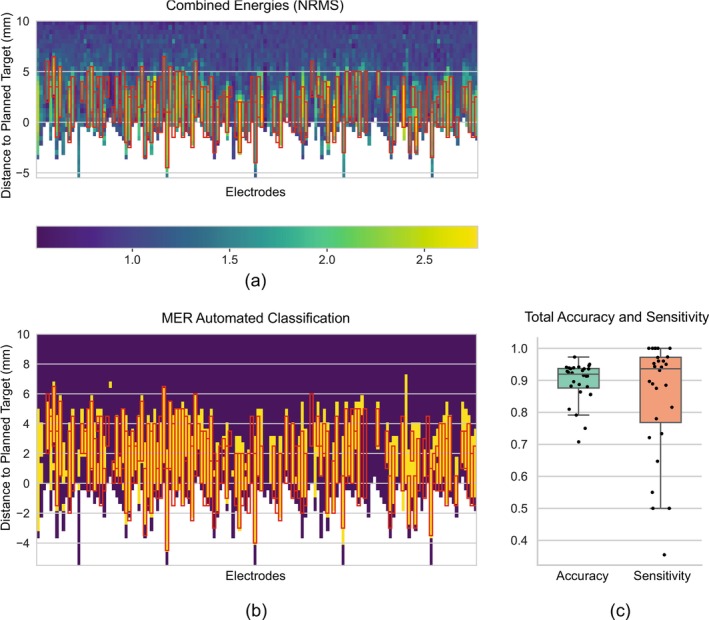
MER energy and automated classification of neural MER Recordings on the Prague‐Multimodal dataset. Results are based on 28 unique trajectories, each comprising five parallel exploration electrodes. (a) Heatmap illustrating normalised energy levels (NRMS) across electrodes and recording positions. The color gradient represents energy values, with red boxes indicating manual labels of STN based on NRMS. (b) Corresponding results from the automated classification of microelectrode recordings (MER): red boxes indicate manually labelled STN boundaries, yellow segments represent STN classified by the algorithm and purple segments represent non‐STN classified by the algorithm. (c) Boxplots summarising the overall performance of the classification algorithm in terms of accuracy (mean = 0.90) and sensitivity (mean = 0.85), with medians, interquartile ranges and outliers displayed.

In the automated MER classification task, evaluated on the *MER‐test* dataset, our model achieved an accuracy of 0.90, a sensitivity of 0.85 and a specificity of 0.91 in the test set compared to expert‐based labelling—see results in Figure 2. The classification is closely aligned with manual STN labels, particularly in regions with high levels of spiking activity. While the overall accuracy remains high, the slightly lower sensitivity suggests that certain STN regions may be misclassified as outside, despite being within the STN according to expert judgement.

### 3.3 Electrode‐STN Coalignment Results

**FIGURE 3:**
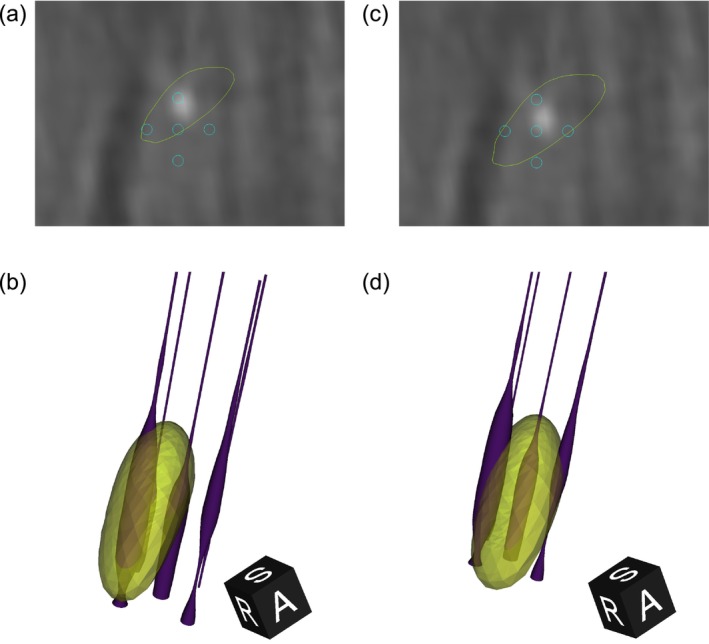
Comparison of electrode placement before and after co‐alignment. (a) 2D slice showing the initial planned electrode positions (blue circles) relative to the subthalamic nucleus (STN) contour (yellow) before co‐alignment. A view along the electrode trajectory with interpolated perpendicular OARM CT cross‐section. (b) 3D view of the STN mesh and electrode trajectories prior to co‐alignment, illustrating initial misalignment. (c) 2D slice after co‐alignment, demonstrating improved alignment of electrodes (blue circles) within the STN contour (yellow). (d) 3D rendering of the STN mesh and electrode trajectories after co‐alignment, illustrating improved spatial alignment. An animated version is available in the online supplement.

In the next step, we applied the optimisation procedure to perform the co‐alignment between the MER and STN surface to the coalignment‐test dataset. Figure 3 illustrates an example case in which the electrode displacement is shown before and after applying the automatic shift correction—shifting the MER electrodes to match their likely position relative to the imaging‐derived segmentation of the STN, positioned according to the surgical plan. Additional subjects of co‐alignment results are available in (Supporting Information S1: Additional co‐alignment Subjects).

**FIGURE 4:**
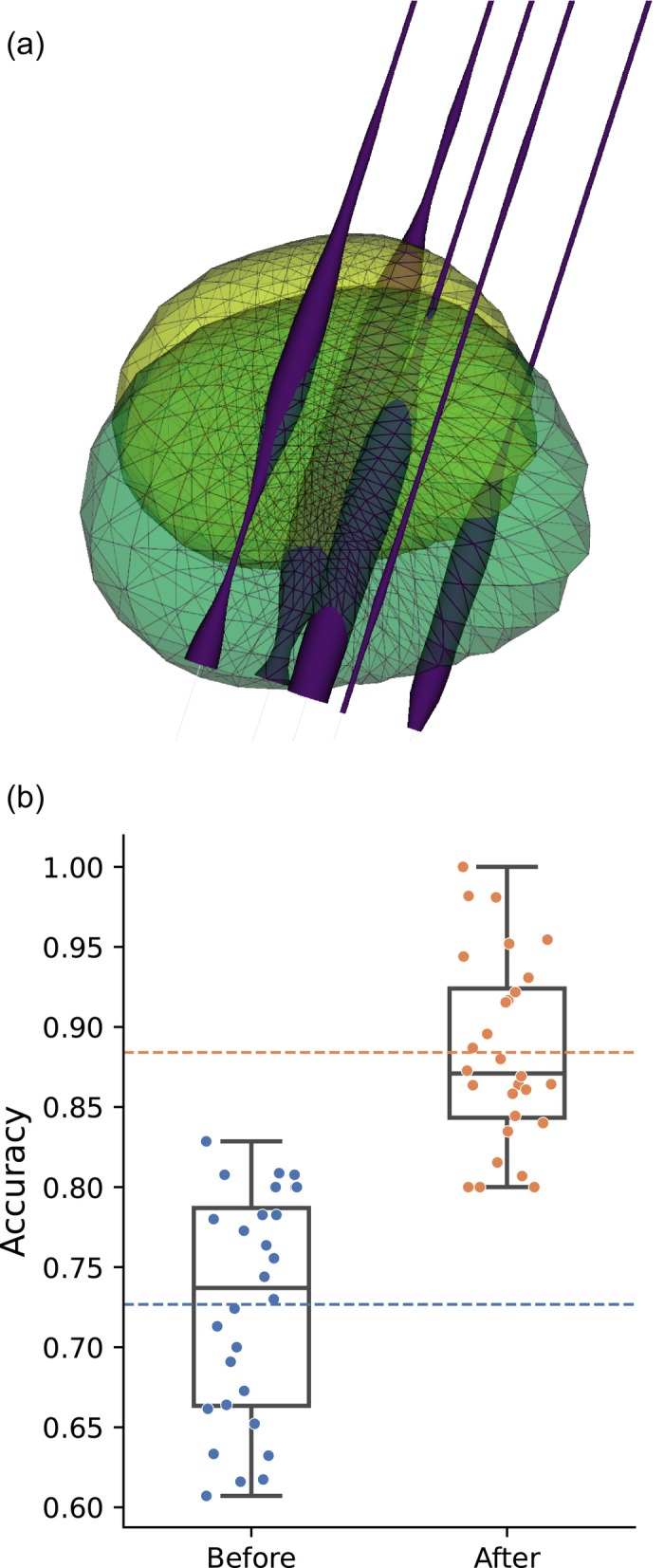
Three‐dimensional visualisation and quantitative evaluation of electrode coalignment in DBS. Results are based on 28 unique trajectories, each comprising five parallel exploration electrodes. (a) A three‐dimensional rendering of the segmented subthalamic nucleus (STN) before (lime green) and after (aquamarine) spatial alignment, with the electrode trajectories shown in purple. (b) The box‐and‐whisker plot compares accuracy of microelectrode placement within STN before and after the STN shift. Overall, these images illustrate how spatial realignment improves the electrodes' placement within the adjusted STN.

Moreover, the developed module supports an alternative approach that involves translation and scaling of the segmented STN shape to better match the electrode recordings. This is illustrated in Figure 4a.

To evaluate the co‐alignment procedure, we applied the pipeline to the *coalignmenttest* dataset with a maximum electrode translation of 3 mm and STN isotropic scaling constrained to ±20%. By convention, we shift the electrodes (native space) and scale the STN mesh. Given that no absolute ground truth exists for the positions of either STN or microelectrodes during the surgery, our validation focusses on (i) improved MER–STN match and (ii) reduced lateral discrepancy relative to OARM‐derived trajectories.

The fraction of MER sites located within the imaging‐defined STN increased from 0.72 ± 0.07 to 0.88 ± 0.06 (Figure 4b).

Then, we compared the *mean lateral distance to OARM trajectory*—defined as the average perpendicular distance between each co‐aligned electrode trajectory and its corresponding path identified on intraoperative OARM imaging—across all MER tracks for each subject (see Supporting Information S1: Section S1.2). This metric does not provide the ground truth STN position, but rather quantifies changes in electrode location relative to a consistent intraoperative anatomical reference. Additionally, the accuracy of coalignment along the trajectories (i.e., direction of the ‘depth’) could not be analysed because the electrode tip was not reliably visible in the OARM scans. Using manually refined STN labels, the mean lateral distance to OARM trajectory across 17 cases decreased by 0.3 mm after co‐alignment, from 1.2 ± 0.6 mm to 0.9 ± 0.5 mm (*p <* 0.02). Overview of individual shift and scaling is available in Supporting Information S1: Section S3.3.

We thus evaluated all individual components of the processing pipeline, as well as finally the full process, using the multimodal dataset.

## 4 Discussion

Building on the Lead‐OR platform, our DBS Co‐alignment toolbox improves the understanding of the spatial relationship between recorded MER signals and the STN by incorporating co‐alignment transformations. To achieve this, we perform segmentation of the STN from preoperative MRI, with the possibility of manual refinement, automatically identify the MER as STN or non‐STN and then perform a co‐alignment to refine anatomical placement information of the electrode in real time.

Although the automatic STN segmentation was not the primary focus of this work, the proposed method achieved a Dice similarity score of approximately 0.6. This closely matches the performance of pBrains (Manjón et al. 2020) when evaluated on our dataset, which also yielded a Dice score of about 0.6—substantially lower than the values originally reported by its authors. This suggests that pBrains does not outperform our approach under the same imaging conditions, and that the discrepancy is likely attributable to differences in acquisition protocols and data quality. For example, the dataset used in (Manjón et al. 2020) offers higher in‐plane resolution and thinner slices (0.74 × 0.74 × 0.4 mm) compared to our dataset (0.95 × 0.95 × 2 mm). In (Li et al. 2016) the authors reported mean Dice scores of 0.86 (left) and 0.88 (right) using a level set method on data with voxel size 0.47 × 0.47 × 3 mm. Visser et al. (2016) implemented a multimodal segmentation method evaluated on 7T MRI with 0.5 mm isotropic resolution and achieved a Dice score of approximately 0.6, while inter‐rater overlap in their study was around 0.7. These observations indicate that higher Dice scores reported in the literature are often associated with superior acquisition protocols and higher‐quality imaging, as well as low agreement between human raters in delineating the STN boundaries.

While T1‐weighted contrast may suffice for initial segmentation, typically using atlas coregistration, achieving patient‐specific accuracy requires T2‐weighted imaging to capture finer anatomical details. In particular, the STN exhibits low contrast against adjacent structures on T1‐weighted scans, whereas T2‐weighted sequences enhance boundary visibility due to differences in tissue relaxation times and sensitivity to iron content (Ewert et al. 2018; Keuken et al. 2014). As our segmentation model was trained using automated labels from the pBrains algorithm rather than manual annotations, this may have introduced a potential bias.

To enhance accuracy and reliability, expert‐labelled data is essential for validation and refinement. Unfortunately, such datasets are currently scarce due to the time‐consuming and labour‐intensive nature of manual annotation. Enhancing STN segmentation would further improve our co‐alignment results. To support this, we offer open‐access segmentation resources within 3D Slicer (source code available at: https: //github.com/IVarha/slicer_dbs_modules , also available in 3D Slicer extension repository as *DBSCoalignment*’) and recommend manual refinement using tools such as WarpDrive (Oxenford et al. 2024) when needed.

For the MER classification, we used the RMS feature provided by Lead‐OR. Our model achieved an accuracy of 0.90, which is slightly better, but comparable to the accuracy of 0.85 reported by others (Karthick et al. 2020) to classify recordings as inside or outside the STN. Other studies have explored different feature sets and classification approaches. For example, a model incorporating 25 features, including both spike‐dependent and spike‐independent characteristics, combined with an ensemble tree classifier, achieved 0.94 precision in STN identification (Coelli et al. 2021). Although such approaches may further refine classification performance, processing a large number of features in real‐time remains a complex computational challenge.

In terms of existing co‐localisation approaches, the authors of (Lourens et al. 2013) fitted an STN atlas to MER positions and reported an accuracy of 0.93 in placing MER sites within the atlas‐defined STN, which is comparable to our reported value of 0.88. However, their approach allowed for 10 mm of displacement, whereas our model limits shift to a maximum of 3 mm. This constraint aligns more closely with clinical observations, as shifts exceeding 4 mm (Khan et al. 2008) are rarely observed in surgeries and can often be mitigated with proper electrode placement techniques (Wu et al. 2022; Petersen et al. 2010). Additionally, our method does not incorporate rotations, which are not among the typical deviations observed during surgery, further constraining the alignment process. Similarly, Luján et al. (2009) demonstrated that their optimisation‐based atlas fit outperformed manual expert fits and AC/PC scaling, achieving 0.81 precision in aligning MER sites with the atlas. Both methods rely on atlas‐based fitting, whereas our approach provides a more flexible, patient‐specific refinement by directly correcting the MRI‐MER misalignment. Furthermore, our study incorporates a comparison with intraoperative O‐arm CT imaging, providing an objective reference to evaluate alignment accuracy.

The 0.3 mm reduction in mean lateral distance after co‐alignment—measured relative to OARM‐derived trajectories—together with the improved spatial agreement between imaging‐defined STN and MER‐defined trajectories (Figure 4b) provides an evaluation of our framework. The human STN measures approximately 6 mm mediolaterally, with the motor territory confined to the dorsolateral/posterolateral portion rather than a fixed linear width. Ultra‐high‐field parcellation studies show that the motor zone comprises roughly half of the STN volume and is spatially compact (Plantinga et al. 2018). Even submillimeter deviations can alter the position of active contacts in relation to adjacent structures, potentially affecting both the therapeutic window and the likelihood of stimulation‐induced side effects (Hamani et al. 2017). As intraoperative targeting decisions are primarily guided by MER‐defined STN borders, improved spatial alignment of MER‐STN, as provided by our tool, can support more precise electrode placement when multiple trajectories are considered.

However, STN segmentation accuracy remained a critical factor that influenced precision, highlighting the need for high‐quality imaging. Specifically, optimised high‐resolution T2 sequences should significantly reduce placement uncertainty, further improving the alignment process. A crucial aspect of our study is the reliance on manual refinement in cases where automatic segmentation did not perform well. When applying the co‐alignment procedure to automatically segmented STN surfaces, we did not observe a statistically significant improvement in electrode displacement. This may indicate limitations in our dataset, including the relatively small number of subjects available for analysis. Expanding the study to multiple centres could provide a more diverse and reliable dataset, ultimately improving the generalisability of our findings.

Although our analysis was performed postoperatively, the Lead‐OR extension is designed for real‐time use in the operating room. Demonstrating an actual change in surgical decision‐making would require a dedicated clinical study, which was beyond the scope of this work. In standard practice, the trajectory for permanent implantation is chosen as the one with a long MER‐defined STN segment close to the assumed STN motor subregion. When multiple trajectories have similar STN inclusion lengths, spatial context within the STN may guide selection. We illustrate this in Supporting Information S1: Co‐alignment example with comparable MER‐based STN coverage showing three trajectories with comparable MER‐based STN coverage but different spatial relationships to the STN.

Establishing a definitive ground truth for shift estimation remains challenging due to multiple sources of error in clinical data. Differences in imaging modalities (OARM, T1, T2, MRI), co‐registration inaccuracies, intraoperative brain shift, electrode bending and postsurgical electrode migration all contribute to uncertainty. Furthermore, deviations between the planned and actual electrode trajectories, as observed in postoperative imaging, suggest that mechanical factors during insertion may introduce further variability. As a result, our toolbox is best used as a guidance tool to assess electrode placement within the STN, which does not require manual intraoperative assessment of the MERs.

## 5 Conclusion

This study introduces a robust and user‐friendly visualisation and processing toolbox that integrates MER data with patient‐specific anatomical information obtained from MRI. Our approach leverages a transformer encoder‐based classification model to process NRMS features extracted from MER signals, enhancing the precision of electrode placement. Powell's optimisation method is used to align electrophysiological recordings with segmented STN, improving spatial accuracy in targeting DBS. This multimodal framework provides valuable information to support accurate and informed decision‐making during surgery.

Designed for researchers and clinicians, our toolbox enables seamless co‐alignment of MER and MRI data, effectively addressing critical challenges in DBS electrode targeting.

Although initial results are encouraging, a validation in multiple clinical centres is essential to thoroughly evaluate the robustness of our approach, especially in accounting for intraoperative brain shifts and variability in electrode placement. Future work should prioritise the integration of clinical outcome measures, enhancement of methodological precision and the execution of prospective clinical trials to validate long‐term clinical effectiveness.

## Author Contributions

**Igor Varga:** conceptualization, formal analysis, investigation, methodology, software, visualization, writing – original draft. **Daniel Novak:** funding acquisition, supervision, writing – review and editing. **Dusan Urgosik:** data curation, resources, validation. **Jan Kybic:** methodology, writing – review and editing. **Filip Ruzicka:** data curation, resources, writing – review and editing. **Robert Jech:** data curation, funding acquisition. **Andreas Horn:** investigation, methodology, writing – review and editing. **Eduard Bakstein:** conceptualization, project administration, supervision, visualization, writing – review and editing.

## Ethics Statement

This study was performed in accordance with the Declaration of Helsinki. This human study was approved by the Ethics Committee of the General University Hospital in Prague under number 59/18. All adult participants provided written informed consent to participate in this study.

## Conflicts of Interest

The authors declare no conflicts of interest.

## Supporting information


**Figure S1:** Architecture of Convolution neural network used for Center prediction and Segmentation. The last layer predicts coefficients of PCA vectors, defining shape primitives and centroid coordinates. These are further converted into 3D mesh vertices.
**Figure S2:** Illustration of the method used for measuring the distance between trajectories. (a) 3D visualisation of the planned electrode trajectory (red line) and the labelled positions (dots) within the segmented anatomical structure. (b) Axial slice of intraoperative OARM imaging, showing the electrode label location (red dot) vs planned (Blue circle). (c) Synthetic example demonstrating the planned (blue line), OARM‐labelled points (green scatter points) and aligned trajectory (orange dashed line) obtained through a least squares fit. The solid black line indicates the shortest distance (1.627 mm) between the aligned and planned trajectories.
**Figure S3:** Supplementary figure showing examples of STN segmentation results on multiple subjects. Each pair of left and right hemisphere images illustrates the segmentation contours overlaid on axial MRI slices, demonstrating variability in shape and anatomical consistency.
**Figure S4:** Comparison of Dice similarity, sensitivity and precision for left and right hemispheres in the IXI and OASIS‐3 datasets. Boxplots show the distribution of each metric across subjects, stratified by hemisphere. The top row corresponds to the IXI dataset, where hemispheres were determined from filename annotations, and the bottom row corresponds to the OASIS‐3 dataset, where metrics were reported separately for left and right segmentations. These plots allow visual comparison of segmentation performance between hemispheres within each dataset, highlighting potential asymmetries and dataset‐specific patterns.
**Figure S5:** Co‐alignment results for three representative subjects. For each subject, axial OARM projection with a bright artifact visible in the position of the central electrode (first and third column) and 3D views of electrode trajectories and segmented STN surfaces (second and fourth column) are shown before and after applying the automatic co‐alignment procedure.
**Figure S6:** Example case illustrating potential intraoperative use of the co‐alignment tool when multiple trajectories show similar MER‐based STN coverage. The yellow mesh is the imaging‐defined STN; purple volumes are MER‐classified STN segments along the Central, Medial and Anterior trajectories, all after coalignment. While STN inclusion lengths are comparable, their spatial positions within the STN differ, which could influence trajectory selection.
**Figure S7:** Distributions of spatial transformations applied during co‐alignment. (Left) Euclidean shift magnitude across all subjects. (Middle) Axis‐specific shifts in x, y and z directions. (Right) Scaling factors applied to the STN mesh.

**Data S1:** Supporting information.

## Data Availability

The data cannot be made publicly available upon publication because they are owned by a third party and the terms of use prevent public distribution. The data that support the findings of this study are available upon reasonable request from the authors.

## References

[ejn70309-bib-0001] Avants, B. B. , N. J. Tustison , J. Wu , P. A. Cook , and J. C. Gee . 2011. “An Open Source Multivariate Framework for *n*‐Tissue Segmentation With Evaluation on Public Data.” Neuroinformatics 9, no. 4: 381–400. 21373993 10.1007/s12021-011-9109-y PMC3297199

[ejn70309-bib-0002] Baniasadi, M. , M. V. Petersen , J. Gon¸calves , et al. 2022. “DBSegment: Fast and Robust Segmentation of Deep Brain Structures Considering Domain Generalization.” Human Brain Mapping 44, no. 2: 762–778. 36250712 10.1002/hbm.26097 PMC9842883

[ejn70309-bib-0003] Coelli, S. , V. Levi , J. Del Vecchio Del Vecchio , et al. 2021. “An Intra‐Operative Feature‐Based Classification of Microelectrode Recordings to Support the Subthalamic Nucleus Functional Identification During Deep Brain Stimulation Surgery.” Journal of Neural Engineering 18, no. 1: 016003. 10.1088/1741-2552/abcb15 33202390

[ejn70309-bib-0004] Dormont, D. , K. G. Ricciardi , D. Tand´e , et al. 2004. “Is the Subthalamic Nucleus Hypointense on T2‐Weighted Images? A Correlation Study Using MR Imaging and Stereotactic Atlas Data.” American Journal of Neuroradiology 25, no. 9: 1516–1523. 15502130 PMC7976405

[ejn70309-bib-0005] Ewert, S. , P. Plettig , N. Li , et al. 2018. “Toward Defining Deep Brain Stimulation Targets in Mni Space: A Subcortical Atlas Based on Multimodal Mri, Histology and Structural Connectivity.” NeuroImage 170: 271–282. 28536045 10.1016/j.neuroimage.2017.05.015

[ejn70309-bib-0006] Fedorov, A. , R. Beichel , J. Kalpathy‐Cramer , et al. 2012. “3D Slicer as an Image Computing Platform for the Quantitative Imaging Network.” Magnetic Resonance Imaging 30, no. 9: 1323–1341. 22770690 10.1016/j.mri.2012.05.001 PMC3466397

[ejn70309-bib-0007] Grabner, G. , A. L. Janke , M. M. Budge , D. Smith , J. Pruessner , and D. L. Collins . 2006. “Symmetric Atlasing and Model Based Segmentation: An Application to the Hippocampus in Older Adults.” In Medical Image Computing and Computer‐Assisted Intervention – MICCAI 2006, edited by D. Hutchison , T. Kanade , J. Kittler , J. M. Kleinberg , F. Mattern , J. C. Mitchell , M. Naor , O. Nierstrasz , C. Pandu Rangan , B. Steffen , M. Sudan , D. Terzopoulos , D. Tygar , M. Y. Vardi , G. Weikum , R. Larsen , M. Nielsen , and J. Sporring , vol. 4191, 58–66. Springer Berlin Heidelberg, Berlin, Heidelberg Series Title: Lecture Notes in Computer Science. 10.1007/11866763_8 17354756

[ejn70309-bib-0008] Gross, R. E. , P. Krack , M. C. Rodriguez‐Oroz , A. R. Rezai , and A.‐L. Benabid . 2006. “Electrophysiological Mapping for the Implantation of Deep Brain Stimulators for Parkinson's Disease and Tremor.” Movement Disorders 21, no. S14: S259–S283. 16810720 10.1002/mds.20960

[ejn70309-bib-0009] Guo, T. , A. G. Parrent , and T. M. Peters . 2007. “Surgical Targeting Accuracy Analysis of Six Methods for Subthalamic Nucleus Deep Brain Stimulation.” Computer Aided Surgery 12, no. 6: 325–334. 18066948 10.3109/10929080701730987

[ejn70309-bib-0010] Hamani, C. , G. Florence , H. Heinsen , et al. 2017. “Subthalamic Nucleus Deep Brain Stimulation: Basic Concepts and Novel Perspectives.” Eneuro 4, no. 5: ENEURO.0140–17.2017. 10.1523/ENEURO.0140-17.2017 PMC5617209 28966978

[ejn70309-bib-0011] Isensee, F. , P. F. Jaeger , S. A. A. Kohl , J. Petersen , and K. H. Maier‐Hein . 2021. “NnU‐Net: A Self‐Configuring Method for Deep Learning‐Based Biomedical Image Segmentation.” Nature Methods 18, no. 2: 203–211. 33288961 10.1038/s41592-020-01008-z

[ejn70309-bib-0012] Karthick, P. A. , K. R. Wan , A. S. An Qi , J. Dauwels , and N. K. K. King . 2020. “Automated Detection of Subthalamic Nucleus in Deep Brain Stimulation Surgery for Parkinson's Disease Using Microelectrode Recordings and Wavelet Packet Features.” Journal of Neuroscience Methods 343: 108826. 32622981 10.1016/j.jneumeth.2020.108826

[ejn70309-bib-0013] Keuken, M. C. , P. L. Bazin , L. Crown , et al. 2014. “Quantifying Inter‐Individual Anatomical Variability in the Subcortex Using 7T Structural MRI.” NeuroImage 94: 40–46. 24650599 10.1016/j.neuroimage.2014.03.032

[ejn70309-bib-0014] Khan, M. F. , K. Mewes , R. E. Gross , and O. Skrinjar . 2008. “Assessment of Brain Shift Related to Deep Brain Stimulation Surgery.” Stereotactic and Functional Neurosurgery 86, no. 1: 44–53. 17881888 10.1159/000108588

[ejn70309-bib-0015] Kocabicak, E. , S. H. Tan , and Y. Temel . 2012. “Deep Brain Stimulation of the Subthalamic Nucleus in Parkinson's Disease: Why so Successful?” Surgical Neurology International 3, no. 5: 312–S314. 10.4103/2152-7806.103024 PMC3514921 23230535

[ejn70309-bib-0016] LaMontagne, P. J. , T. L. Benzinger , J. C. Morris , et al. 2019. “OASIS‐3: Longitudinal Neuroimaging, Clinical, and Cognitive Dataset for Normal Aging and Alzheimer Disease.” ISSN: 1901‐4902 Pages: 2019.12.13.19014902.

[ejn70309-bib-0017] Li, B. , C. Jiang , L. Li , J. Zhang , and D. Meng . 2016. “Automated Segmentation and Reconstruction of the Subthalamic Nucleus in Parkinson's Disease Patients.” Neuromodulation: Technology at the Neural Interface 19, no. 1: 13–19. 26484724 10.1111/ner.12350

[ejn70309-bib-0018] Lourens, M. , H. Meijer , M. Contarino , et al. 2013. “Functional Neuronal Activity and Connectivity Within the Subthalamic Nucleus in Parkinson's Disease.” Clinical Neurophysiology 124, no. 5: 967–981. 23182834 10.1016/j.clinph.2012.10.018

[ejn70309-bib-0019] Lozano, C. S. , M. Ranjan , A. Boutet , et al. 2018. “Imaging Alone Versus Microelectrode Recording–Guided Targeting of the STN in Patients With Parkinson's Disease.” Journal of Neurosurgery 130, no. 6: 1847–1852. 30074454 10.3171/2018.2.JNS172186

[ejn70309-bib-0020] Luján, J. L. , A. M. Noecker , C. R. Butson , et al. 2009. “Automated 3‐Dimensional Brain Atlas Fitting to Microelectrode Recordings From Deep Brain Stimulation Surgeries.” Stereotactic and Functional Neurosurgery 87, no. 4: 229–240. 19556832 10.1159/000225976 PMC2836941

[ejn70309-bib-0021] Manjón, J. V. , A. Bertó , J. E. Romero , et al. 2020. “pBrain: A Novel Pipeline for Parkinson Related Brain Structure Segmentation.” NeuroImage: Clinical 25: 102184. 31982678 10.1016/j.nicl.2020.102184 PMC6992999

[ejn70309-bib-0022] Montgomery, E. B., Jr. 2012. “Microelectrode Targeting of the Subthalamic Nucleus for Deep Brain Stimulation Surgery.” Movement Disorders 27, no. 11: 1387–1391. 22508394 10.1002/mds.25000

[ejn70309-bib-0023] Moran, A. , I. Bar‐Gad , H. Bergman , and Z. Israel . 2006. “Real‐Time Refinement of Subthalamic Nucleus Targeting Using Bayesian Decision‐Making on the Root Mean Square Measure.” Movement Disorders 21, no. 9: 1425–1431. 16763982 10.1002/mds.20995

[ejn70309-bib-0024] Oxenford, S. , A. S. R´ıos , B. Hollunder , et al. 2024. “Warpdrive: Improving Spatial Normalization Using Manual Refinements.” Medical Image Analysis 91: 103041. 38007978 10.1016/j.media.2023.103041 PMC10842752

[ejn70309-bib-0025] Oxenford, S. , J. Roediger , C. Neudorfer , et al. 2022. “Lead‐OR: A Multimodal Platform for Deep Brain Stimulation Surgery.” eLife 11: e72929. 35594135 10.7554/eLife.72929 PMC9177150

[ejn70309-bib-0026] Park, S.‐C. , J. K. Lee , S. M. Kim , E. J. Choi , and C. S. Lee . 2018. “Systematic Stereotactic Error Reduction Using a Calibration Technique in Single‐Brain‐Pass and Multitrack Deep Brain Stimulations.” Operative Neurosurgery 15, no. 1: 72–80. 28961863 10.1093/ons/opx183

[ejn70309-bib-0027] Petersen, E. A. , E. M. Holl , I. Martinez‐Torres , et al. 2010. “Minimizing Brain Shift in Stereotactic Functional Neurosurgery.” Operative Neurosurgery 67, no. 3: ons213–ons221. 10.1227/01.NEU.0000380991.23444.08 20679927

[ejn70309-bib-0028] Plantinga, B. R. , Y. Temel , Y. Duchin , et al. 2018. “Individualized Parcellation of the Subthalamic Nucleus in Patients With Parkinson's Disease With 7T MRI.” NeuroImage 168: 403–411. 27688203 10.1016/j.neuroimage.2016.09.023 PMC5479742

[ejn70309-bib-0029] Powell, M. J. D. 1964. “An Efficient Method for Finding the Minimum of a Function of Several Variables Without Calculating Derivatives.” Computer Journal 7, no. 2: 155–162.

[ejn70309-bib-0030] Reinacher, P. C. , B. Varkuti , M. T. Kruger , et al. 2019. “Automatic Segmentation of the Subthalamic Nucleus: A Viable Option to Support Planning and Visualization of Patient‐Specific Targeting in Deep Brain Stimulation.” Operative Neurosurgery 17, no. 5: 497–502. 30860266 10.1093/ons/opz015

[ejn70309-bib-0031] Schafer, A. , B. U. Forstmann , J. Neumann , et al. 2012. “Direct Visualization of the Subthalamic Nucleus and Its Iron Distribution Using High‐Resolution Susceptibility Mapping.” Human Brain Mapping 33, no. 12: 2831–2842. 21932259 10.1002/hbm.21404 PMC6870400

[ejn70309-bib-0032] Schlaier, J. R. , C. Habermeyer , J. Warnat , et al. 2011. “Discrepancies Between the MRI‐ and the Electrophysiologically Defined Subthalamic Nucleus.” Acta Neurochirurgica 153, no. 12: 2307–2318. 21744142 10.1007/s00701-011-1081-7

[ejn70309-bib-0033] Temel, Y. , P. Wilbrink , A. Duits , et al. 2007. “Single Electrode and Multiple Electrode Guided Electrical Stimulation of the Subthalamic Nucleus in Advanced Parkinson's Disease.” Operative Neurosurgery 61, no. 5: 346–357. 10.1227/01.neu.0000303993.82149.98 18091250

[ejn70309-bib-0034] Thompson, J. A. , S. Oukal , H. Bergman , et al. 2018. “Semi‐Automated Application for Estimating Subthalamic Nucleus Boundaries and Optimal Target Selection for Deep Brain Stimulation Implantation Surgery.” Journal of Neurosurgery 130, no. 4: 1224–1233. 29775152 10.3171/2017.12.JNS171964

[ejn70309-bib-0035] Varga, I. , E. Bakstein , G. Gilmore , J. May , and D. Novak . 2024. “Statistical Segmentation Model for Accurate Electrode Positioning in Parkinson's Deep Brain Stimulation Based on Clinical Low‐Resolution Image Data and Electrophysiology.” PLoS ONE 19, no. 3: e0298320 Publisher: Public Library of Science. 38483943 10.1371/journal.pone.0298320 PMC10939223

[ejn70309-bib-0036] Vaswani, A. , N. Shazeer , N. Parmar , et al. 2023. “Attention Is All You Need.” arXiv:1706.03762.

[ejn70309-bib-0037] Verhagen, R. , P. R. Schuurman , P. van den Munckhof , M. F. Contarino , R. M. A. de Bie , and L. J. Bour . 2016. “Comparative Study of Microelectrode Recording‐Based STN Location and MRI‐Based STN Location in Low to Ultra‐High Field (7.0 T) T2‐Weighted MRI Images.” Journal of Neural Engineering 13, no. 6: 066009. 27739406 10.1088/1741-2560/13/6/066009

[ejn70309-bib-0038] Visser, E. , M. C. Keuken , B. U. Forstmann , and M. Jenkinson . 2016. “Automated Segmentation of the Substantia Nigra, Subthalamic Nucleus and Red Nucleus in 7 T Data at Young and Old Age.” NeuroImage 139: 324–336. 27349329 10.1016/j.neuroimage.2016.06.039 PMC4988791

[ejn70309-bib-0039] Wu, Y.‐X. , W. Xiang , J.‐J. Wang , et al. 2022. “A Modified Dura Puncture Procedure to Reduce Brain Shift in Deep Brain Stimulation Surgery: One Institution's Experience.” Frontiers in Neurology 13: 845926. 35295828 10.3389/fneur.2022.845926 PMC8920348

[ejn70309-bib-0040] Yushkevich, P. A. , J. Piven , H. C. Hazlett , et al. 2006. “User‐Guided 3D Active Contour Segmentation of Anatomical Structures: Significantly Improved Efficiency and Reliability.” NeuroImage 31, no. 3: 1116–1128. 16545965 10.1016/j.neuroimage.2006.01.015

[ejn70309-bib-0041] Zrinzo, L. 2019. “Letter: Systematic Stereotactic Error Reduction Using a Calibration Technique in Single‐Brain‐Pass and Multitrack Deep Brain Stimulations.” Operative Neurosurgery 16, no. 2: E67. 10.1093/ons/opy317 30496586

