# Supplementary material for: Precise Electrode Co‐Alignment in Deep Brain Stimulation Fusing Neuroimaging and Electrophysiology
Source: Eur J Neurosci. 2025 Nov 19;62(10):e70309. doi: 10.1111/ejn.70309 (PMC12629890; doi:10.1111/ejn.70309)
Supplement: Supplementary file 1 — Figure S1: Architecture of Convolution neural network used for Center prediction and Segmentation. The last layer predicts coefficients of PCA vectors, defining shape primitives and centroid coordinates. These are further converted into 3D mesh vertices. Figure S2: Illustration of the method used for measuring the distance between trajectories. (a) 3D visualisation of the planned electrode trajectory (red line) and the labelled positions (dots) within the segmented anatomical structure. (b) Axial slice of intraoperative OARM imaging, showing the electrode label location (red dot) vs planned (Blue circle). (c) Synthetic example demonstrating the planned (blue line), OARM‐labelled points (green scatter points) and aligned trajectory (orange dashed line) obtained through a least squares fit. The solid black line indicates the shortest distance (1.627 mm) between the aligned and planned trajectories. Figure S3: Supplementary figure showing examples of STN segmentation results on multiple subjects. Each pair of left and right hemisphere images illustrates the segmentation contours overlaid on axial MRI slices, demonstrating variability in shape and anatomical consistency. Figure S4: Comparison of Dice similarity, sensitivity and precision for left and right hemispheres in the IXI and OASIS‐3 datasets. Boxplots show the distribution of each metric across subjects, stratified by hemisphere. The top row corresponds to the IXI dataset, where hemispheres were determined from filename annotations, and the bottom row corresponds to the OASIS‐3 dataset, where metrics were reported separately for left and right segmentations. These plots allow visual comparison of segmentation performance between hemispheres within each dataset, highlighting potential asymmetries and dataset‐specific patterns. Figure S5: Co‐alignment results for three representative subjects. For each subject, axial OARM projection with a bright artifact visible in the position of the central electrode (first and [file EJN-62-0-s002.pdf]

Supplementary Material

This supplementary material accompanies the manuscript titled *"Precise Electrode Co-alignment in Deep Brain Stimulation Fusing Neuroimaging and Electrophysiology"*, authored by Igor Varga, Daniel Novak, Simon Oxenford, Dusan Urgosik, Jan Kybic, Filip Ruzicka, Pavel Filip, Robert Jech, Andreas Horn, and Eduard Bakstein.

S1. Supplementary methods

S1.1. CNN Architecture

The CNN architecture, used for segmentation, is shown in figure S1.

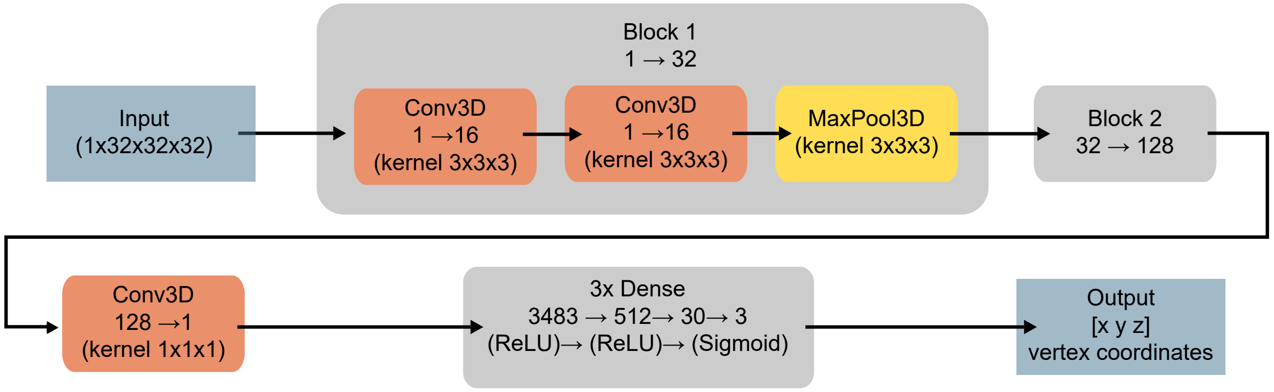

Figure S1: Architecture of Convolution neural network used for Center prediction and Segmentation. The last layer predicts coefficients of PCA vectors, defining shape primitives, and centroid coordinates. These are further converted into 3D mesh vertices.

S1.2. Measurement of Distance Between Trajectories

To quantify the displacement between trajectories, we first constructed an optimal fitted line through the labelled points identified on intraoperative OARM images. This line was generated using a least-squares approach, with the constraint that it must remain parallel to the planned electrode trajectory - as shown in Figure S2.

After determining this optimal alignment, we computed the shortest Euclidean distance between the aligned OARM-labelled trajectory and the original planned trajectory. This measurement provided the average local displacement error reported in our validation analysis.

Due to the procedure of OARM measurement and the inability to locate the exact electrode tip (microelectrode sheath is visible in the OARM CT image but not the MER tip), we could not compute the exact displacement between the OARM an the MER.

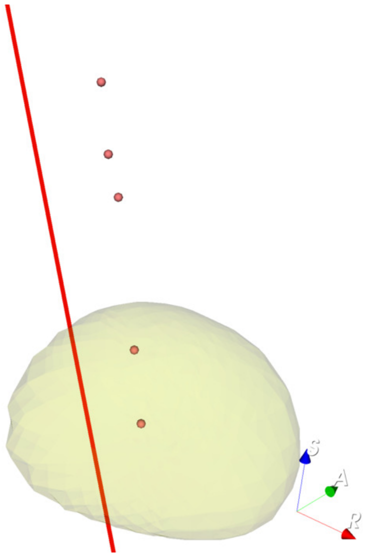

(a)

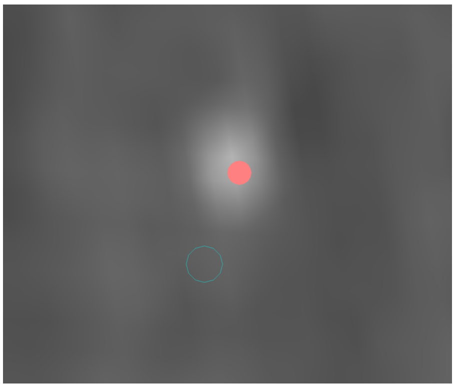

(b)

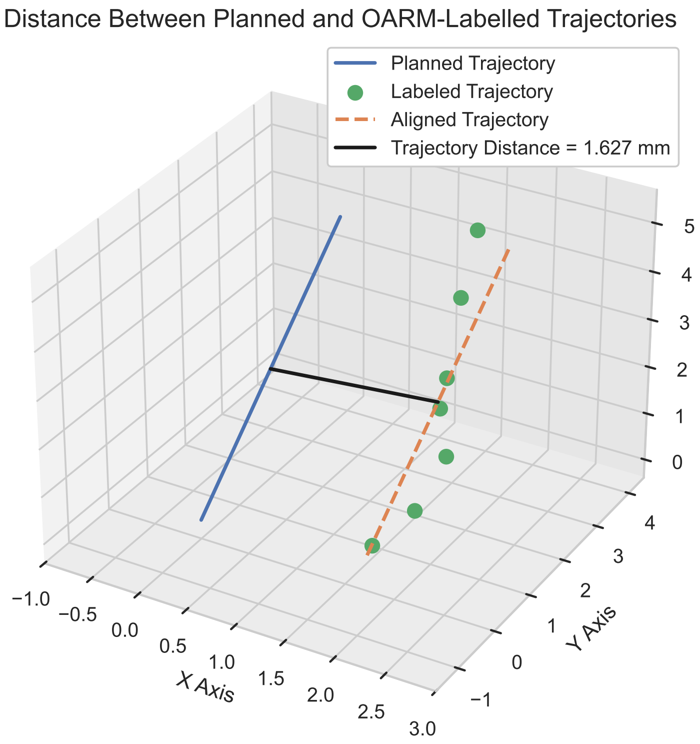

(c)

Figure S2: Illustration of the method used for measuring the distance between trajectories. (a) 3D visualisation of the planned electrode trajectory (red line) and the labelled positions (dots) within the segmented anatomical structure. (b) Axial slice of intraoperative OARM imaging, showing the electrode label location (red dot) vs planned (Blue circle). (c) Synthetic example demonstrating the planned (blue line), OARM-labelled points (green scatter points), and aligned trajectory (orange dashed line) obtained through a least squares fit. The solid black line indicates the shortest distance (1.627 mm) between the aligned and planned trajectories.

S1.3. Algorithm to Compute Optimisation Criterion

Algorithm 1: Calculation of the Optimisation Criterion $C(\mathbf{x})$ for Electrode Alignment

---

Input x:

- Original electrode positions (*orig-points*)
- Inside/outside STN automatic classification labels (*in-out*)
- Shift vector [x,y,z] for electrodes (*shift*)
- Scaling factor for the STN mesh (*scaling*)
- STN mesh (*mesh*)

Output: Alignment error $C(\mathbf{x})$, representing the discrepancy between electrode positions and the STN mesh.

Step 1: Apply Electrode Shift
Adjust the positions of the electrodes by subtracting the shift vector (*shift*) from the original electrode positions (*orig_points*).

Step 2: Scale the STN Mesh
Transform the STN mesh (*mesh*) by applying the scaling factor (*scaling*) to adjust its dimensions proportionally.

Step 3: Determine Shifted Relative Recording Positions
Classify each shifted electrode position as inside or outside the scaled STN mesh based on its spatial relationship to the transformed mesh.

Step 4: Generate Alignment Weights
Compare the STN/NON-STN classification labels assigned to each MER recording location (*in-out*) with its spatial position relative to the STN mesh after shifting the electrodes and scaling the mesh. If an electrode classified as STN is located outside the scaled STN mesh or an electrode classified as NON-STN is located inside the scaled STN mesh after shifting, assign a weight of 1 (indicating misalignment). Otherwise, assign a weight of 0 (indicating correct alignment).

Step 5: Compute Distances to the Transformed Mesh
Calculate the minimum Euclidean distance from each shifted electrode position to the nearest point on the surface of the transformed STN mesh.

Step 6: Calculate Weighted Alignment Error
Compute the alignment error by multiplying the weights computed in Step 4 by the distances from Step 5 and summing the results. The final error metric is obtained by averaging over all electrode positions, providing a measure of overall alignment quality.

Step 7: Return the Result
Output the alignment error $C(\mathbf{x})$, which quantifies the discrepancy between the adjusted electrode positions and the transformed STN mesh.

S2. Segmentation Results and Examples

S2.1. Segmentation Examples

The Figure S3 shows contours, resulting from the automatic STN segmentation.

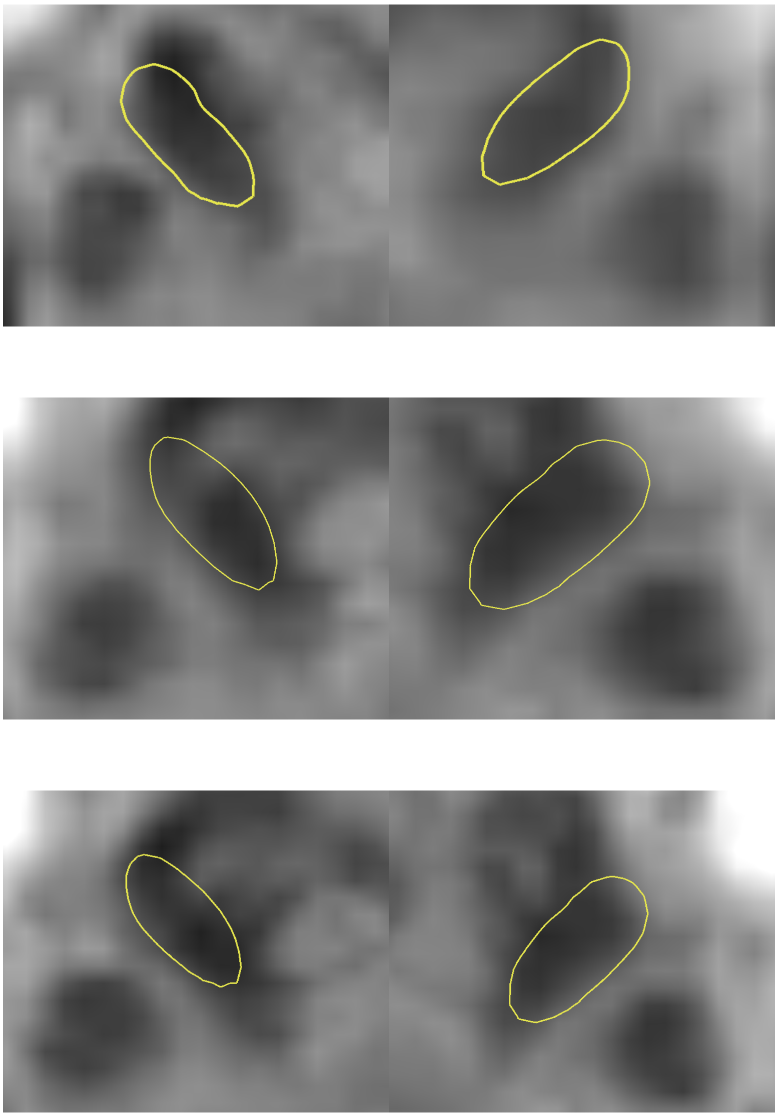

Figure S3: Supplementary figure showing examples of STN segmentation results on multiple subjects. Each pair of left and right hemisphere images illustrates the segmentation contours overlaid on axial MRI slices, demonstrating variability in shape and anatomical consistency.

S2.2. Distribution of Segmentation Metrics

The Figure S4 shows additional breakdown of segmentatation metrics by hemisphere.

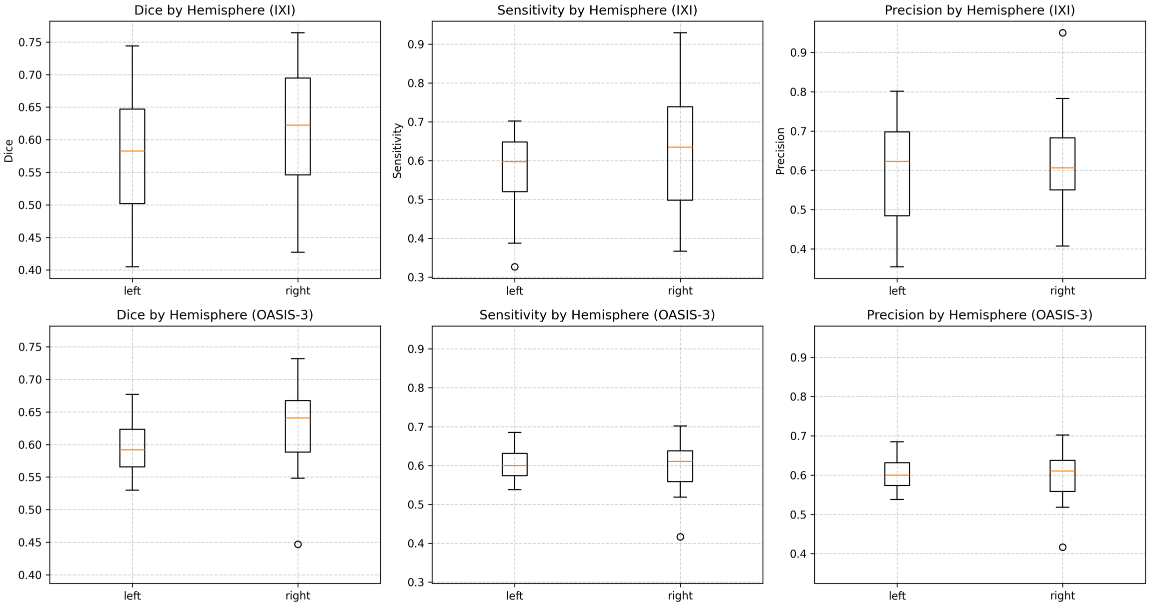

Figure S4: Comparison of Dice similarity, sensitivity, and precision for left and right hemispheres in the IXI and OASIS-3 datasets. Boxplots show the distribution of each metric across subjects, stratified by hemisphere. The top row corresponds to the IXI dataset, where hemispheres were determined from filename annotations, and the bottom row corresponds to the OASIS-3 dataset, where metrics were reported separately for left and right segmentations. These plots allow visual comparison of segmentation performance between hemispheres within each dataset, highlighting potential asymmetries and dataset-specific patterns.

S3. Co-alignment Results and Examples

S3.1. Additional Co-alignment Examples

Three additional examples of co-alignment results together with the OARM data are shown in Figure S5.

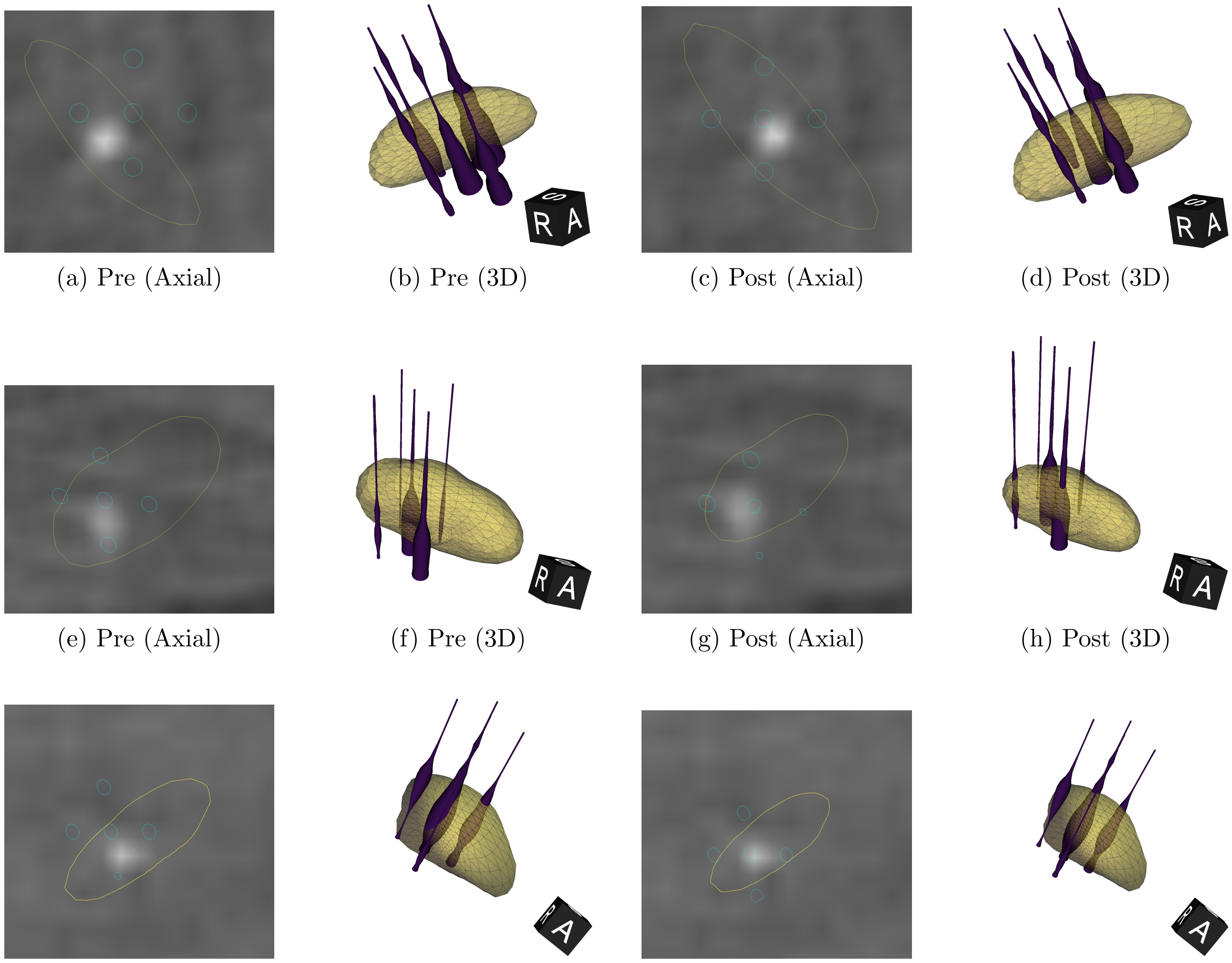

(a) Pre (Axial) (b) Pre (3D) (c) Post (Axial) (d) Post (3D)

(e) Pre (Axial) (f) Pre (3D) (g) Post (Axial) (h) Post (3D)

(i) Pre (Axial) (j) Pre (3D) (k) Post (Axial) (l) Post (3D)

Figure S5: Co-alignment results for three representative subjects. For each subject, axial OARM projection with a bright artifact visible in the position of the central electrode (first and third column) and 3D views of electrode trajectories and segmented STN surfaces (second and fourth column) are shown before and after applying the automatic co-alignment procedure.

S3.2. Co-alignment example with comparable MER-based STN coverage

This example illustrates a potential intraoperative scenario in which the co-alignment tool could aid trajectory selection when multiple candidate tracks have similar MER-based STN inclusion lengths. Figure S6 shows three trajectories intersecting the STN (Central, Medial, Anterior) represented by their MER-classified STN segments (dark purple volumes) in relation to the co-aligned, imaging-defined STN (yellow mesh). All three trajectories have comparable MER-derived STN coverage; however, their spatial positions within the STN differ. While the current study was performed on postoperative data, this example demonstrates how, in a real-time intraoperative setting, the co-alignment visualisation could assist the surgeon in selecting the trajectory that maximises coverage of the dorsolateral STN.

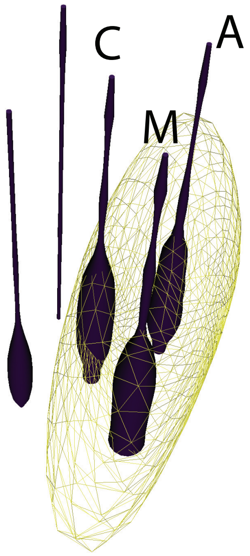

Figure S6: Example case illustrating potential intraoperative use of the co-alignment tool when multiple trajectories show similar MER-based STN coverage. The yellow mesh is the imaging-defined STN; purple volumes are MER-classified STN segments along the Central, Medial, and Anterior trajectories, all after coalignment. While STN inclusion lengths are comparable, their spatial positions within the STN differ, which could influence trajectory selection.

S3.3. Observed shift and scaling ranges

To quantify the transformations applied during co-alignment, we computed: (1) the Euclidean shift magnitude across all axes, (2) individual axis-specific shifts, and (3) scaling factors applied to the STN mesh. Figure S7 illustrate these distributions.

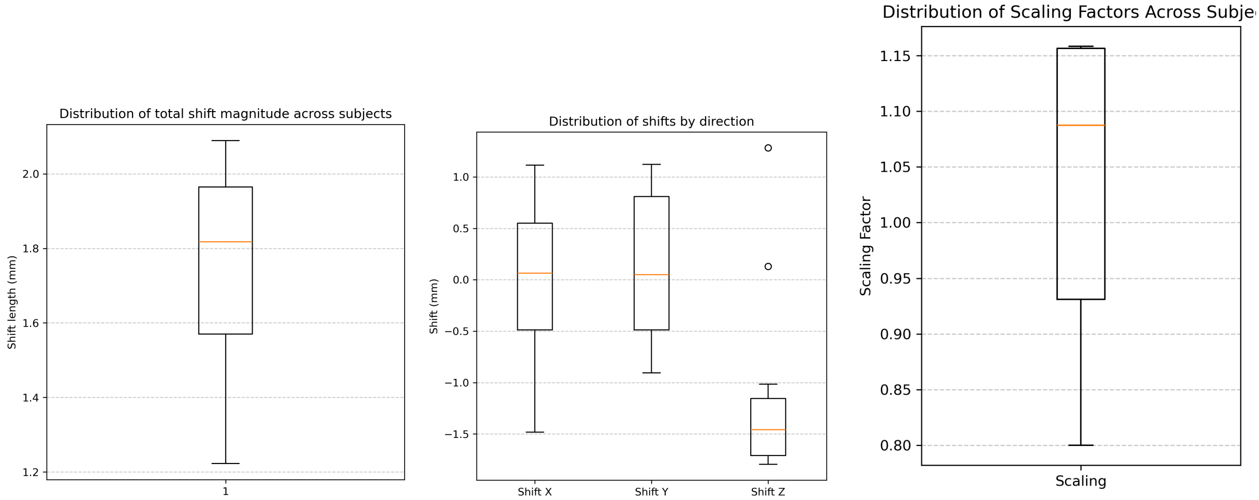

Figure S7: Distributions of spatial transformations applied during co-alignment. (Left) Euclidean shift magnitude across all subjects. (Middle) Axis-specific shifts in x, y, and z directions. (Right) Scaling factors applied to the STN mesh.
